# Association between gallbladder stone disease and prostate cancer: A nationwide population-based study

**DOI:** 10.18632/oncotarget.9062

**Published:** 2016-04-27

**Authors:** Chien-Hua Chen, Cheng-Li Lin, Chia-Hung Kao

**Affiliations:** ^1^ Digestive Disease Center, Show-Chwan Memorial Hospital, Changhua, Taiwan; ^2^ Department of Food Science and Technology, Hung Kuang University, Taichung, Taiwan; ^3^ Chung Chou University of Science and Technology, Yuanlin Township, Changhua, Taiwan; ^4^ Management Office for Health Data, China Medical University Hospital, Taichung, Taiwan; ^5^ College of Medicine, China Medical University, Taichung, Taiwan; ^6^ Graduate Institute of Clinical Medical Science, School of Medicine, College of Medicine, China Medical University, Taichung, Taiwan; ^7^ Department of Nuclear Medicine and PET Center, China Medical University Hospital, Taichung, Taiwan

**Keywords:** metabolic disorder, gallbladder stone disease, prostate cancer

## Abstract

**Objectives:**

Chronic inflammation and abnormal cholesterol metabolism are involved in the pathogenesis of gallbladder stone disease (GSD) and that of prostate cancer in experimental studies. We assessed the association between GSD and prostate cancer in this population-based study.

**Results:**

The cumulative incidence of prostate cancer (log-rank test: *P* <.001) and the risk of prostate cancer (1.64 vs 1.14 per 10 000 person-y, adjusted hazard ratio [aHR] = 1.30, 95% confidence interval [CI] = 1.22-1.39) were greater in the patients with GSD than in those without GSD. Furthermore, the risk of prostate cancer increased with the time of follow-up after a diagnosis of GSD, particularly after 9 years of follow-up (aHR = 1.95, 95% CI = 1.74-2.19).

**Materials and Methods:**

We identified 9496 patients who were diagnosed with GSD between 1998 and 2011 from Taiwan's Longitudinal Health Insurance Database 2000 as the study cohort. We randomly selected 37 983 controls from the non-GSD population and used frequency matching by age, sex, and index year for the control cohort. All patient cases were followed until the end of 2011 to measure the incidence of prostate cancer.

**Conclusion:**

GSD is associated with an increased risk of prostate cancer, and the risk increases with the time of follow-up after a diagnosis of GSD.

## INTRODUCTION

Prostate cancer is the most common cancer in men in Western countries. The incidence and prevalence of prostate cancer has steadily increased worldwide, and it accounted for 28% of newly diagnosed cancers in American men in 2010 and 22.2% of newly diagnosed cancers in European men in 2008 [1, 2]. The incidence of prostate cancer is much lower for Asian men, but the reported incidence for Asian migrant men has increased from 10–20 per 100 000 person-years in their native lands to approximately 50 per 100 000 person-years in the United States [3]. Furthermore, the incidence of prostate cancer for Taiwanese men has increased from 7.5/100 000 in 1992 to 23.5/100 000 in 2007 [4].

Gallbladder stone disease (GSD) is a worldwide disease; its prevalence increases with socioeconomic development, and it affects approximately 10% of the adult population in Western countries and 5% of the adult population in Taiwan [5]. Furthermore, the prevalence of GSD is expected to increase with the increasing incidence of the metabolic syndrome, lifestyle westernization worldwide, and widespread use of ultrasound in clinical practice [6, 7].

Experimental studies have shown both chronic inflammation and abnormal cholesterol metabolism are involved in the pathogenesis of GSD and that of prostate cancer [7–10, 11, 12]. The association between GSD and prostate cancer has even been supported in one ecology study and one case-control study, respectively [13, 14]. The close association between GSD and prostate cancer may raise the concern about the development of prostate cancer after the diagnosis of GSD, a common clinical scenario, and portend more studies for clarifying the real explanation for this association. However, the association between GSD and the development of prostate cancer was only mentioned in a Japanese population-based cohort study [8]. In this study, we hypothesized that a history of GSD might be associated with an increased risk of prostate cancer. We conducted a nationwide population-based cohort study by analyzing data from the Longitudinal Health Insurance Database 2000 (LHID2000) of Taiwan to assess the association between GSD and the subsequent development of prostate cancer.

## RESULTS

We selected 9496 patients with GSD as the case cohort and 37 983 patients without GSD as the control cohort (Table 1). The mean age of the GSD cohort was 56.4 years, the mean age of the non-GSD cohort was 55.7 years, and approximately 62.6% of the patients were aged ≥50 years. The occupations of approximately 48.1% of the patients were white-collar, and more than half of the patients lived in urbanized areas (level ≥2). The patients in the GSD cohort were more likely to have comorbidities and a history of using antihypertensive agents than those in the non-GSD cohort (*P* < .001). Figure 2 illustrates that the cumulative incidence of prostate cancer in the GSD cohort was significantly higher than in the non-GSD cohort during the mean follow-up of 6.55 years for the GSD cohort and 6.60 years for the non-GSD cohort (*P* <.001).

**Table 1:** Comparison of demographics and comorbidity between gallbladder stone disease patients and controls

	Gallbladder stone disease (N =9496)		Control (N =37983)		p-value
	n	(%)	n	(%)	
Age, year					0.99
≤ 49	3555	(37.4)	14220	(37.4)	
50-64	2779	(29.3)	11116	(29.3)	
≥65	3162	(33.3)	12647	(33.3)	
Mean (SD)^#^	56.4	(15.8)	55.7	(16.1)	0.0002
Occupation					0.01
White collar	4566	(48.1)	18081	(47.6)	
Blue collar	3206	(33.8)	13412	(35.3)	
Others^‡^	1724	(18.2)	6490	(17.1)	
Urbanization level^§^					0.0001
1 (highest)	2730	(28.8)	10664	(28.1)	
2	2883	(30.4)	10854	(28.6)	
3	1667	(17.6)	6959	(18.3)	
4(lowest)	2216	(23.3)	9506	(25.0)	
Comorbidity					
Hyperlipidemia	2483	(26.2)	6386	(16.8)	<0.001
Diabetes	1279	(13.5)	3190	(8.40)	<0.001
Hypertension	3995	(42.1)	12315	(32.4)	<0.001
BPH	655	(6.90)	1682	(4.43)	<0.001
Urinary stones	769	(8.10)	1575	(4.15)	<0.001
Urinary tract infection	355	(3.74)	1060	(2.79)	<0.001
Obesity	105	(1.11)	212	(0.56)	<0.001
Asthma	767	(8.08)	2184	(5.75)	<0.001
CAD	2286	(24.1)	5855	(15.4)	<0.001
COPD	1664	(17.5)	4798	(12.6)	<0.001
Stroke	563	(5.93)	1814	(4.78)	<0.001
Medication					
Antihypertensive medications	3542	(37.3)	10633	(28.0)	<0.001

Chi-square test;

# T-test

§ The urbanization level was categorized by the population density of the residential area into 4 levels, with level 1 as the most urbanized and level 4 as the least urbanized.

‡ Other occupations included primarily retired, unemployed, or low income populations.

**Figure 1:**
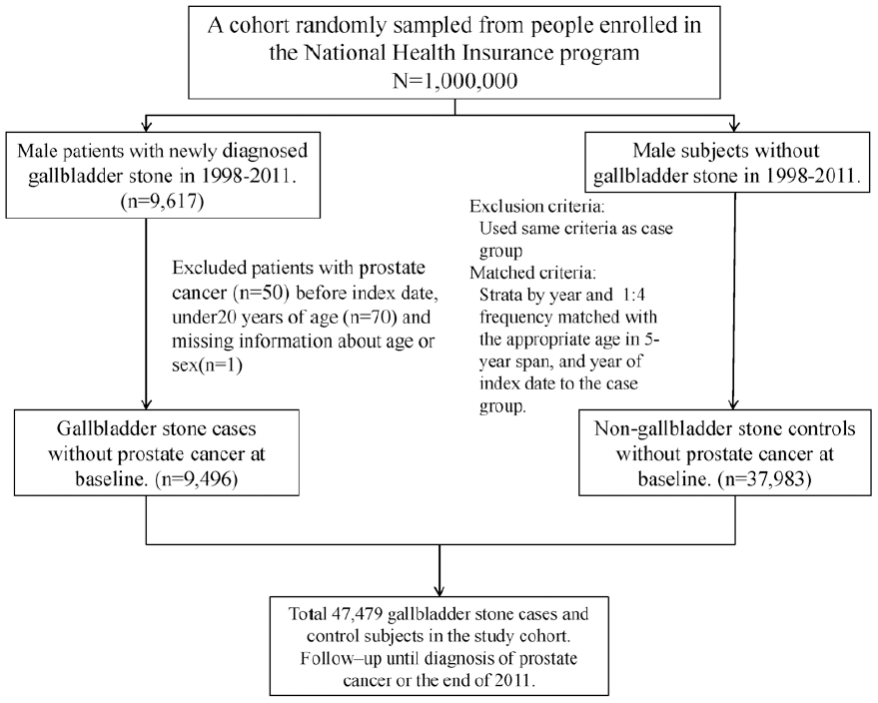
A flow chart to summarize the study design and the subjects' selection in this study

**Figure 2:**
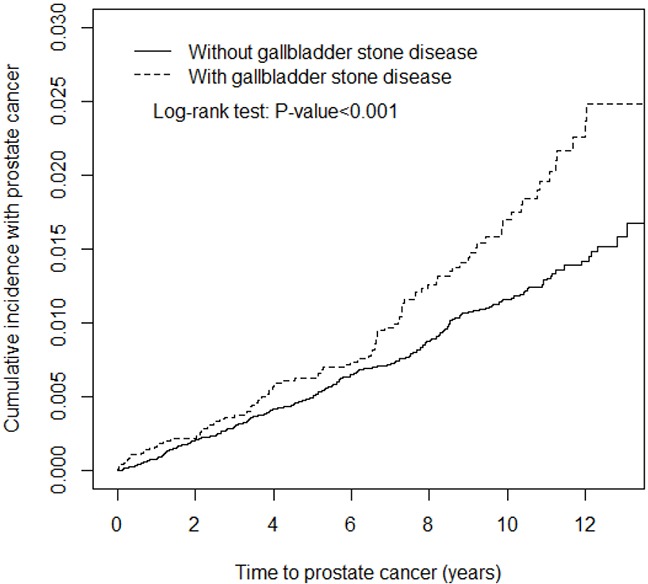
Kaplan-Meir method determined cumulative incidence of Prostate cancer compared between gallbladder stone cohorts and comparisons without gallbladder stone disease

The incidence density rates of prostate cancer were 1.64 and 1.14 per 10 000 person-years for the GSD and non-GSD cohorts, respectively (Table 2). The risk of prostate cancer was significantly higher in the GSD cohort than in the non-GSD cohort (adjusted HR [aHR] = 1.30; 95% CI = 1.22-1.39). The patients with GSD had a significantly increased risk of prostate cancer compared with the patients without GSD in all age groups (aHR = 1.45, 95% CI = 1.33-1.58 for the patients aged ≤64 y and aHR = 1.24, 95% CI = 1.11-1.38 for those aged >64 y). The occupation-specific analyses showed that the patients with GSD had a significantly higher risk of prostate cancer than those without GSD did, except for the white-collar workers. The risk of prostate cancer in the patients with GSD, when stratified by urbanization level and comorbidities, including antihypertensive medication use, was also higher than in those without GSD.

**Table 2:** Comparison of incidence densities of Prostate cancer between patients with and without gallbladder stone disease stratified by demographic characteristics and comorbidity

	Gallbladder stone disease							
	Yes			No			Crude HR (95% CI)	Adjusted HR^†^ (95% CI)
	Event	PY	Rate^#^	Event	PY	Rate^#^		
All	102	62229	1.64	285	250766	1.14	1.44(1.35, 1.54)***	1.30(1.22, 1.39)***
Age								
≤ 64	26	43992	0.59	59	177925	0.33	1.78(1.64, 1.94)***	1.45(1.33, 1.58)***
>64	76	18237	4.17	226	72841	3.10	1.34(1.20, 1.50)***	1.24(1.11, 1.38)***
Occupation								
White collar	31	30421	1.02	111	119133	0.93	1.09(0.98, 1.22)	1.01(0.92, 1.12)
Blue collar	36	20758	1.73	106	89949	1.18	1.47(1.31, 1.65)***	1.29(1.16, 1.45)***
Others^‡^	35	11051	3.17	68	41684	1.63	1.94(1.68, 2.24)***	1.69(1.47, 1.94)***
Urbanization level^§^								
1 (highest)	33	17989	1.83	95	71073	1.34	1.37(1.21, 1.56)***	1.26(1.12, 1.42)***
2	26	19067	1.36	77	72203	1.07	1.28(1.12, 1.46)***	1.19(1.06, 1.35)**
3	15	10949	1.37	43	45417	0.95	1.45(1.23, 1.70)***	1.28(1.10, 1.49)**
4(lowest)	28	14224	1.97	70	62073	1.13	1.75(1.53, 1.99)***	1.55(1.36, 1.75)***
Comorbidity^&^								
No	12	24291	0.49	72	143810	0.50	0.99(0.87, 1.12)	1.19(1.09, 1.30)***
Yes	90	37938	2.37	213	106956	1.99	1.19(1.09, 1.30)***	1.31(1.21, 1.43)***
Medication								
Antihypertensive medications								
No	38	42019	0.90	137	192110	0.71	1.27(1.16, 1.39)***	1.27(1.17, 1.38)***
Yes	64	20210	3.17	148	58656	2.52	1.26(1.12, 1.40)***	1.31(1.17, 1.46)***

# Rate incidence rate, per 10,000 person-years; Crude HR represented relative hazard ratio;

† Adjusted HR multivariable analysis including age, occupation, urbanization level, comorbidity of hyperlipidemia, diabetes, hypertension, BPH, urinary stones, urinary tract infection, obesity, asthma, CAD, COPD, stroke and antihypertensive medications;

§ The urbanization level was categorized by the population density of the residential area into 4 levels, with level 1 as the most urbanized and level 4 as the least urbanized.

‡ Other occupations included primarily retired, unemployed, or low income populations.

& Comorbidity Only to have one of comorbidities (including hyperlipidemia, diabetes, hypertension, BPH, urinary stones, urinary tract infection, obesity, asthma, CAD, COPD, and stroke) classified as the comorbidity group p<0.05,

** p<0.01,

*** p<0.001

The risk of prostate cancer (aHR = 1.30, 95% CI = 1.22-1.39) was greater for the GSD cohort than for the non-GSD cohort after adjustment for age, occupation, urbanization level, and comorbidities of hyperlipidemia, diabetes, hypertension, BPH, urinary stones, urinary tract infection, obesity, asthma, CAD, COPD, stroke, and antihypertensive medication use in Cox proportional hazards regression (Table 3). The risk of developing prostate cancer increased with age (aHR = 1.09, 95% CI = 1.08-1.09 for every 1 y). The patients with white-collar and blue-collar jobs had a higher risk of developing cancer compared with those with other occupations. Compared with patients living in the least urbanized areas, patients living in the most urbanized areas had a higher risk of developing prostate cancer (aHR = 1.40, 95% CI = 1.04-1.89). The risk of developing prostate cancer was greater for patients with comorbidities of hyperlipidemia (aHR = 1.42, 95% CI = 1.12-1.79) and BPH (aHR = 1.49, 95% CI = 1.02-2.19).

**Table 3:** Hazard ratios of Prostate cancer in association with age, occupation, urbanization level, and comorbidities in univariable and multivariable Cox regression models

Variable	Crude		Adjusted^†^	
	HR	(95%CI)	HR	(95%CI)
Gallbladder stone disease	1.44	(1.35, 1.54)***	1.30	(1.22, 1.39)***
Age, years	1.09	(1.08, 1.09)***	1.09	(1.08, 1.09)***
Occupation				
White collar	1	(reference)	1.36	(1.04, 1.78)*
Blue collar	1.35	(1.26, 1.45)***	1.33	(1.01, 1.76)*
Others^‡^	2.06	(1.91, 2.22)***	1	(reference)
Urbanization level^§^				
1 (highest)	1.12	(1.03, 1.21)**	1.40	(1.04, 1.89)*
2	0.88	(0.81,0.95)**	1.20	(0.89, 1.61)
3	0.80	(0.73, 0.88)***	1.04	(0.74, 1.46)
4(lowest)	1	(reference)	1	(reference)
Baseline co-morbidities (no vs yes)				
Hyperlipidemia	2.09	(1.96, 2.23)***	1.42	(1.12, 1.79)**
Diabetes	1.56	(1.41, 1.71)***	0.80	(0.57, 1.11)
Hypertension	3.38	(3.19, 3.59)***	1.11	(0.86, 1.42)
BPH	3.92	(3.52, 4.35)***	1.49	(1.02, 2.19)*
Urinary stones	1.28	(1.12, 1.47)***	1.13	(0.71, 1.80)
Urinary tract infection	2.00	(1.73, 2.31)***	1.05	(0.64, 1.74)
Obesity	1.66	(1.18, 2.33)**	1.45	(0.46, 4.55)
Asthma	2.03	(1.83, 2.25)***	0.94	(0.65, 1.37)
CAD	3.33	(3.14, 3.54)***	1.24	(0.98, 1.57)
COPD	2.59	(2.41, 2.77)***	0.95	(0.73, 1.24)
Stroke	1.75	(1.54, 1.98)***	0.69	(0.44,1.06)
Medication				
Antihypertensive medications	3.60	(3.39, 3.81)***	1.19	(0.93, 1.53)

Crude HR represented relative hazard ratio;

† Adjusted HR represented adjusted hazard ratio: mutually adjusted for age, occupation, urbanization level, comorbidity of hyperlipidemia, diabetes, hypertension, BPH, urinary stones, urinary tract infection, obesity, asthma, CAD, COPD, stroke and antihypertensive medications in Cox proportional hazard regression.

* p < 0.05,

** p < 0.01,

*** p < 0.001

Regarding the trends in prostate cancer development after a GSD diagnosis, the risk of developing prostate cancer increased progressively with an incremental duration of follow-up, particularly after 9 years of follow-up (aHR = 1.95, 95% CI = 1.74-2.19) (Table 4).

**Table 4:** Trends of prostate cancer risks stratified by follow-up years of gallbladder stone disease

Follow time, years	Gallbladder stone disease							
	Yes			No			Crude HR (95% CI)	Adjusted HR^†^ (95% CI)
	Event	PY	Rate^#^	Event	PY	Rate^#^		
≤3	32	25180	1.27	99	100860	0.98	1.29(1.20, 1.40)***	1.16(1.08, 1.25)***
4-6	23	18493	1.24	87	74557	1.17	1.07(0.97, 1.17)	0.89(0.56, 1.42)
7-9	29	12077	2.40	66	48949	1.35	1.78(1.62, 1.96)***	1.73(1.58, 1.90)***
>9	18	6479	2.78	33	26401	1.25	2.22(1.98, 2.50)***	1.95(1.74, 2.19)***

# Rate incidence rate, per 10,000 person-years; Crude HR represented relative hazard ratio;

† Adjusted HR multivariable analysis including age, occupation, urbanization level, comorbidity of hyperlipidemia, diabetes, hypertension, BPH, urinary stones, urinary tract infection, obesity, asthma, CAD, COPD, stroke and antihypertensive medications;

* p<0.05,

** p<0.01,

*** p<0.001

## DISCUSSION

We observed that 62.6% of the patients with GSD were older than 50 (mean: 56.4 ± 15.8) years, and GSD was more prevalent in the patients with white-collar jobs (48.1%) and in those living in ≥ level 2 urbanized areas (59.2%). The aging process is accompanied by increased biliary cholesterol secretion, decreased activity of cholesterol 7 α hydroxylase, decreased bile salt synthesis, impaired gallbladder motility, and increased exposure time to lithogenic factors [21, 22]. Westernized dietary habits and sedentary lifestyles may explain why GSD was more prevalent in the subjects with white-collar jobs and living in more urbanized areas [6, 23].

Our results suggest the patients with GSD had more comorbidities. A low serum high density lipoprotein cholesterol (HDL-C) concentration can increase biliary cholesterol secretion, whereas a high triglyceride level can result in supersaturated bile and impaired gallbladder emptying [24, 25]. Insulin resistance with hyperinsulinemia can increase biliary cholesterol secretion, and hyperglycemia can impair both hepatic bile acid secretion and gallbladder emptying [26, 27]. Hypertension can impair gallbladder contractility by activating the sympathetic nervous system [28]. Circulating inflammatory cytokines induced by BPH, urinary stones, and urinary tract infection may increase the risk of GSD [29]. Obesity can enhance hepatic cholesterol synthesis and biliary cholesterol secretion [30]. In addition to smoking that can reduce the secretion of prostaglandin and mucus in the gallbladder, chronic inflammation from both asthma and COPD can also increase the risk of GSD [7, 28]. The association between GSD and cardiovascular disease (CVD) is due to cholesterol supersaturation in GSD and cholesterol deposition in atherosclerotic plaque [31, 32].

According to our analysis, increased prostate cancer risk was associated with GSD, aging, white-collar and blue-collar jobs, the highest urbanization level (level 1), hyperlipidemia, and BPH (Table 3). Dysplastic lesions require decades to evolve into prostate cancer, and the reported incidence of prostate cancer for men has increased 100 folds from fourth decade to seventh decade [33, 34]. Westernized dietary habits and sedentary lifestyles may explain why prostate cancer was more prevalent in the subjects with white- and blue-collar jobs and living in the most urbanized areas as high meat intake can induce structural DNA damage in the prostate and physical activity can decrease low-grade inflammation to reduce prostate carcinogenesis [35, 36]. Cholesterol over-accumulation in the cell membrane may facilitate procarcinogenic cell signaling in the prostate [37, 38]. Aging is a common risk factor for BPH and prostate cancer. In addition to local inflammation in the prostate that may induce carcinogenesis, patients with BPH may seek more medical consultations and thus have more opportunities for prostate cancer screening [4, 39].

GSD and prostate cancer may be associated because of the common risk factors. Nevertheless, our results reveal that GSD is related to subsequent prostate cancer development after adjustment for age, occupation, urbanization level, hyperlipidemia, diabetes, hypertension, BPH, urinary stones, urinary tract infection, obesity, asthma, CAD, COPD, stroke, and antihypertensive medication use in Cox proportional hazards regression. Moreover, our results also suggest that the risk of prostate cancer increases with an incremental duration of follow-up after GSD diagnosis, particularly after 9 years of follow-up (Table 4). We could not ascertain the causal relationship between GSD and prostate cancer, but our results support an increased risk of prostate cancer after GSD diagnosis.

Cholesterol metabolism and chronic inflammation are reported to be the major pathophysiological mechanisms for the association between GSD and prostate cancer [8, 9]. Biliary cholesterol supersaturation contributes to the development of GSD, and an abnormal cholesterol metabolism predisposes people to developing prostate cancer by promoting tumor growth through transducing signals, inhibiting apoptotic signals, and stimulating malignant potentials [8, 37, 38]. Moreover, circulating cholesterol is a component of androgen, which can increase the proliferation of prostate cancer cells [9]. GSD can activate chronic inflammation in the body by repeatedly irritating the gallbladder mucosa. The influence of cytokine gene polymorphisms on the antitumor immune response and tumor angiogenesis of prostate cancer is supported in the literature [9, 10]. Regarding the chronic inflammatory process, GSD and prostate cancer share several inflammatory genes, such as interleukin-8 and vascular endothelial growth factor A [9, 10]. Interleukin-8 is involved in neutrophil chemotaxis, whereas vascular endothelial growth factor A increases vascular permeability, angiogenesis, cell growth and migration, and apoptosis.

Our study had several strengths. First, it is the largest population-based study on the association between GSD and prostate cancer, and a longitudinal database with a 14-year observation period for a representative cohort of 1 000 000 residents was used to examine the association between GSD and the subsequent development of prostate cancer. Second, the recruited patients were sampled from the approximately 99% of Taiwan residents who are covered by the Taiwan NHI program.

Our study also had several limitations. First, we should acknowledge the evidence derived from this retrospective cohort study is generally of lower methodological evidence than that from randomized controlled trials because a retrospective cohort study is subject to many biases due to lack of the necessary adjustments for possible confounding factors. For adjustment, we used COPD diagnosis, obesity, and occupation and urbanization level to replace smoking habits, BMI, and socioeconomic status, respectively. GSD was consistently related to prostate cancer development according to multivariable Cox proportional hazards regression. However, the association between GSD and prostate cancer requires further studies to ascertain this association is an epiphenomena or a causal relationship. Second, the risk of GSD and prostate cancer might have been underestimated in our study if the patients did not seek medical consultation. However, the NHI program covers more than 99% of Taiwan's population, and the accessibility and affordability of health care are high in Taiwan. Finally, we could not individually review the medical records to validate the diagnosis of GSD and prostate cancer. However, the NHIRD covers a highly representative population of Taiwan because the reimbursement policy is universal and the government is the only buyer. All insurance claims should be scrutinized by medical reimbursement specialists and peer reviewed according to the standard diagnosed criteria in the study. The doctors or hospitals will face a lot of penalties if they made wrong diagnoses or coding. The diagnoses of GSD and prostate cancer based on ICD-9 codes in this study should be highly reliable through the accessibility and affordability of NHI program, high sensitivity and specificity of ultrasound in the diagnosis of GSD, and the linkage to the registry of Catastrophic Illness Certificate. In addition, some related studies about GSD or prostate cancer with the same diagnosed method and criteria by ICD-9 coding were already been published [18, 40].

In conclusion, our population-based cohort study indicates that GSD is associated with an increased risk of developing prostate cancer. The risk of prostate cancer increased with the time of follow-up after a diagnosis of GSD, particularly after 9 years of follow-up. However, further studies are required to clarify whether GSD is a causal risk factor for prostate cancer.

## MATERIALS AND METHODS

### Data source

A retrospective cohort study was conducted using registration and claims data sets from 1998 to 2011 obtained from the LHID2000.The Taiwan National Health Insurance (NHI) program is a universal, single-payer, and compulsory health insurance system with more than 99% population coverage for 23 million Taiwanese residents and more than 97% of the hospitals and clinics contracted (http://www.nhi.gov.tw/english/index.aspx) [15]. The LHID2000 contains comprehensive health care data on ambulatory care claims, inpatient claims, and the prescriptions of 1 000 000 randomly selected insured beneficiaries from National Health Insurance Research Database (NHIRD) enrollment record. The data of NHIRD (http://w3.nhri.org.tw/nhird//date_01.html) are maintained in the National Health Research Institutes (NHRI) (http://nhird.nhri.org.tw/), which is established by the government as a nonprofit foundation. All encrypted data were deposited in an appropriate open and public repository, and the researchers can use the database for research after approval of formal application [16, 17]. The Ethics Review Board of China Medical University and Hospital in Taiwan approved this study (CMUH104-REC2-115). The diseases were coded according to the International Classification of Diseases, Ninth Revision, Clinical Modification (ICD-9-CM) diagnosis codes, 2001 edition.

### Sampled participants

Male patients aged ≥20 years with newly diagnosed GSD (ICD-9-CM codes 574.0, 574.1, 574.2, 574.6, 574.7, 574.8, 574.9) from 1998–2011 were identified using the LHID2000. The date of GSD diagnosis was defined as the index date. Patients with a history of prostate cancer (ICD-9-CM code 185) were excluded. The non-GSD cohort consisted of patients randomly selected from the LHID2000 without a GSD history. For each GSD case, 4 men without GSD were randomly selected as the control cohort through frequency matching according to sex, age (every 5-y span), and the index date year. The same exclusion criteria were also applied to non-GSD controls. Figure 1 provided a flow chart to summarize the study design and the subjects' selection in this study.

### Follow-up and cancer ascertainment

All the study subjects were followed up until they were diagnosed with prostate cancer or censored because of loss to follow-up, withdrawal from the insurance program, death, or the end of 2011. We ascertained the death, loss of follow-up, or withdrawal of NHI program from the NHI withdrawal history files kept in NHIRD. We ascertained the diagnosis of prostate cancer through a record linkage to the registry of Catastrophic Illness Certificate, a part of NHI program in Taiwan [18]. The date of applications for the approved certificate was defined as the index date. The registry of Catastrophic Illness Certificate is integrated from multiple NHI database to provide comprehensive information for the copayment regulation of NHI program. The patients with certain defined diseases, such as cancers, organ transplantation, autoimmune diseases, or end stage renal disease with hemodialysis or peritoneal dialysis, can apply for the Catastrophic Illness Certificate to obtain copayment exemptions from the NHI program. The approval of Catastrophic Illness Certificate for cancers requires cytological or pathological evidence supporting the diagnosis of malignancy.

### Variables of interest

The socioeconomic variables used in this study comprised age, occupation, and urbanization level. Occupation and urbanization level have been well defined in previous studies [19, 20]. In this study, white-collar workers were defined as people with long indoor working hours, such as institutional, business, and industrial administration personnel. By contrast, blue-collar workers were defined as people with long outdoor working hours, such as fishermen, farmers, or industrial laborers. Other occupations included retired, unemployed, or low-income occupations. The levels of urbanization were classified according to population density (people/km^2^), and level 4 indicated the least urbanization. We defined baseline comorbidities, including hyperlipidemia (ICD-9-CM code 272), diabetes (ICD-9-CM code 250), hypertension (ICD-9-CM codes 401-405), BPH (ICD-9-CM code 600), urinary stones (ICD-9-CM codes 590, 595), urinary tract infection (ICD-9-CM codes 592.0, 592.1, 594.0, 594.1), obesity (ICD-9-CM code 278), asthma (ICD-9-CM code 493), coronary artery disease (CAD) (ICD-9-CM codes 410-414), chronic obstructive pulmonary disease (COPD) (ICD-9-CM codes 491, 492, 496), and stroke (ICD-9-CM codes 430-438). A history of antihypertensive medication use was included in the analysis.

### Statistical analysis

The distribution of demographic data (age, occupation, and urbanization level), comorbidities (hyperlipidemia, diabetes, hypertension, BPH, urinary stones, urinary tract infection, obesity, asthma, CAD, COPD, and stroke), and antihypertensive medication use were compared between the GSD and non-GSD cohorts by using a chi-square test to examine categorical variables and a *t* test to analyze continuous variables. The Kaplan–Meier method was used to depict the curves of cumulative prostate cancer incidence for the 2 cohorts, and the log-rank test was employed to examine the difference between the curves. We computed the incidence density rate (per 1000 person-y) of follow-up for each cohort. Univariable and multivariable Cox proportional hazards regression models were used to examine the effect of GSD on the risk of prostate cancer, which was expressed as a hazard ratio (HR) with a 95% confidence interval (CI). The multivariable models were adjusted for age, occupation, urbanization level, and the comorbidities of hyperlipidemia, diabetes, hypertension, BPH, urinary stones, urinary tract infection, obesity, asthma, CAD, COPD, stroke, and antihypertensive medication use. SAS Version 9.3 software (SAS Institute, Cary, NC, USA) was used for data analysis. A significance level of *P* <.05 was used for the comparisons for 2-sided testing.

## Abbreviations

GSD: gallbladder stone disease
aHR: adjusted hazard ratio
CI: confidence interval
LHID2000: Longitudinal Health Insurance Database 2000
NHI: National Health Insurance
NHIRD: National Health Institute Research Database
NHRI: National Health Research Institute
ICD-9-CM: International Classification of Diseases, Ninth Edition, Clinical Modification
BPH: benign prostatic hyperplasia
CAD: coronary artery disease
COPD: chronic obstructive pulmonary disease
HDL-C: high density lipoprotein cholesterol
CVD: cardiovascular disease
BMI: body mass index
